# Characterization of *Klebsiella granulomatis* pathogenic to silkworm, *Bombyx mori* L.

**DOI:** 10.1007/s13205-014-0255-4

**Published:** 2014-10-19

**Authors:** M. K. Mohanta, A. K. Saha, D. K. M. A. Saleh, M. S. Islam, K. S. B. Mannan, M. Fakruddin

**Affiliations:** 1 Department of Zoology, Rajshahi University, Rajshahi, 6205 Bangladesh; 2 Center for Food and Waterborne Diseases, icddr’b, Dhaka, Bangladesh; 3 Institute of Food Science and Technology, Bangladesh Council of Scientific and Industrial Research, Dhaka, Bangladesh

**Keywords:** Silkworm, Pathogenic bacteria, *Klebsiella pneumoniae*, Haemolymph, Antibiotic

## Abstract

Bacterial disease of silkworm causes significant reduction of silk production leading to huge economic loss. This study aims to isolate bacteria from diseased silkworm and to determine its pathogenicity and antibiotic resistance. A strain of *Klebsiella granulomatis* has been isolated from silkworm haemolymph which was later identified on the basis of biochemical tests and 16S rRNA gene sequencing. The optimum culture condition of *K. granulomatis* was determined at pH 7.0 and 37 °C temperature. The strain was resistant to most of the antibiotics used in this study except azithromycin, gentamycin and ciprofloxacin. The strain is capable to reproduce flacherrie like symptoms with high mortality rate when re-injected into healthy silkworm. Treatment with low dose of ciprofloxacin was found to be effective to prevent flacherrie induced by the isolated *K. granulomatis* strain.

## Introduction

Sericulture is an agro-based industry practiced in the greater Rajshahi, Chapai Nawabganj, Natore, Bogra and Naogaon and certain nontraditional areas in Bangladesh. It is grouped under village and small enterprises sector that plays major role for the creation of sustainable employment and income (Ishtiaque et al. 2013). The poor people of the society, the landless, and the poor woman in particular, can be involved in sericulture activities. According to an estimate, livelihood of about 0.1 million people in Rajshahi region is directly or indirectly involved with sericulture industry (Islam et al. 2004).

The mulberry silkworm, *Bombyx mori,* has been domesticated for silk production for more than 5,000 years and provides the major source of income for 30 million families. Geographically, Asia is the main producer of mulberry silk in the world and produces over 98 % of the total global output (Savithri et al. 2013). During the silkworm rearing, the silkworm comes into contact with pathogenic agents (viz. Protozoa-microsporidians, virus, Fungi and Bacteria) which accounts for considerable loss to cocoon production (Samson 1995). Rearing silkworm free from diseases is a major constraint to silkworm rearers (Priyadharshini et al. 2008). About 34–40 % the total crop in a year has been reported to be loss due to diseases (Sheebha et al. 2008).

Bacterial diseases are of common occurrence in Bangladesh. Prevalence of bacterial infection in plant and insect is high due to elevated temperature (above 30 °C) and low relative humidity (below 80 %). Bacterial flacherrie is a common disease of mulberry silkworm (kaito et al. 2002). The aetiology of bacterial diseases is not fully understood because of the multiplicity of bacterial types involved in bacterial infections (Choudhury et al. 2002). Insects infected with pathogenic bacteria exhibit symptoms such as loss of apetite, diarrhea, vomitting, larvae softening and foul odor upon death (Singh et al. 2011, Sakthivel et al. 2012).

From economic point of view, this disease is of particular concern as it's prevalence mostly is in the ripen mounted worms and it cause death within 24 h (Rahmathulla 2012). Since there are no specific preventive measures for the occurrence and spread of disease other than sanitized rearing methods, the only commercial practice today is to discard large stocks of worms in case of infection to avoid the spread of disease (Acharya et al. 2002). Antibiotics are widely used in sericulture industry as a component of bed disinfectants and as therapeutic applications against bacterial diseases (Subramanian et al. 2009). Broad spectrum antibiotics viz., penicillin, streptomycin, tetracycline and chloramphenicol were already tried on silkworm and found successful (Venkatesh and Srivastava 2010). Antibiotics in silkworm are approved for four different purposes: disease treatment, disease prevention, disease control and health maintenance or growth promotion (Phillips et al. 2004).

The aim of this study was to isolate pathogenic bacteria from diseased silkworm, to determine the pathogenicity of the isolated bacterium and to evaluate the therapeutic effects of antibiotics on the pathogen.

## Materials and methods

### Collection of silkworms

Diseased silkworm larvae were collected from Bangladesh Sericulture Research and Training Institute (BSRTI), Rajshahi. They were then used as a source of inocula for the isolation of the pathogenic microorganisms.

### Isolation and characterization of the microbes from the diseased silkworms

The microbes were isolated from silkworm haemolymph. One loopful of haemolymph was directly transferred into nutrient broth media (Hi Media, India), which was incubated for 2 days at 37 °C and subjected to shaking at 120 rpm on an orbital shaker. Control flasks without inoculates were also prepared and incubated at 37 °C with an orbital shaker. The cultures that were found turbid after a period of 0 up to 2 days were used as inocula in subsequent experiments.

### Microscopic examination and identification of bacterial cells

For the identification of the pathogenic bacterium, morphological characterizations, microscopic observations, growth characteristics, biochemical tests and antibiotic sensitivity tests were performed. The microorganisms were identified according to Bergey’s Manual of Systematic Bacteriology (Holt 2005).

### Identification of the pathogen by 16S rRNA gene sequence

Genomic DNA of the bacterial isolate was isolated according to Mohanta et al. (2012). Gene fragments specific for the highly variable region of the bacterial 16S rRNA gene were amplified by PCR using universal PCR primer as described by Loffler et al. (2000) (Sigma, USA) in a thermal cycler (MJ Research Inc., Watertown, USA). The sequence of the forward primer was 16SF 5′-GAGTTTGATCCTGGCTCAG-3′ and the sequence of the reverse primer was 16SR 5′-GAAAGGAGGTGATCCAGCC-3′. The PCR products were subjected to 1 % agarose gel electrophoresis, stained with ethidium bromide and visualized on a UV transilluminator for the presence of about 1,500 bp PCR products. Amplified 16S rRNA gene PCR products were purified using StrataPrep PCR purification kit (Stratagene, USA) according to the manufacturer’s protocol. Sequencing reactions were carried out using ABI-Prism Big dye terminator cycle sequencing ready reaction kit and the PCR products were purified by a standard protocol. The purified cycle sequenced products were analyzed with an ABIPrism 310 genetic analyzer. The chromatogram sequencing files were edited using Chromas 2.32. The homology of the 16S rRNA gene sequences was checked with the 16S rRNA gene sequences of other organisms that had already been submitted to GenBank database using the BLASTN (http://www.ncbi.nih.gov/BLAST/) algorithm.

### Effect of temperature and pH on bacterial growth

Temperature and pH influence bacterial growth. For the effect of pH, culture medium (nutrient broth, Hi-media) was adjusted to pH 5.0, 7.0, and 8.0. Incubation temperature was varied at, 25, 30 and 37 °C. Bacterial cell density of liquid cultures was determined by measuring optical density at 660 nm with photoelectric colorimeter (AE-11 M, Erma Inc., Tokyo) (Mohanta et al. 2012).

### Pathogenicity of the isolate to silkworm

Larvae of hybrid strain *B. mori* were reared at 25 °C. Fresh mulberry leaves (average size: 10, 20 cm) were obtained from a garden of mulberry, Department of Zoology, University of Rajshahi. The isolated bacterium was cultured for 24 h in nutrient broth and harvested by centrifuge and re-suspended in phosphate buffered saline and cell count was determined and diluted with PBS. Fifty healthy newly moulted fifth-instar larvae were included in each experimental group. Mulberry leaves inoculated with appropriate number of the bacterium were fed to the larvae two times a day. Symptoms of the diseases were observed and mortality rate was recorded each day. Statistical analysis was completed using SPSS 16.0, and the median lethal concentration (LC50) and regression equations were obtained.

### Determination of pathogenicity of the isolated bacterium against silkworm

Two treatments were used to investigate the effects of ciprofloxacin on pathogenicity of the isolate against silkworm.

*Treatment 1* Third instars of healthy silkworm larvae were selected for the experiment. Ten larvae from each group were considered for the treatment. Fresh broth culture of the isolated bacterium at 10^7^ cfu mL^−1^ was spread or smeared on mulberry leaves and fed to the 3rd instar larvae two times in a day. The shape, size and weight of the cocoon were recorded.

*Treatment 2* Fresh broth culture of the test bacterium at 10^7^cfu.mL^−1^ were smeared on mulberry leaves and fed to the 3rd instar larvae. In the same time an antibiotic (ciprofloxacin at 20 µl/gbw) was injected into the gut of the larvae with the help of micro injection (needle size 0.30 × 8 mm/30G × 5/16″) two times a day for getting cure of the disease. The treatment was carried out up to pupation of the larvae. The shape, size and weight of the cocoon were recorded.

## Results

### Isolation and identification of the bacterium

Bacteria were isolated by plating onto an agar solidified nutrient medium. The plates were incubated at 37 °C for 2 days and bacterial colonies were found to grow on the medium. Results of microscopic analysis of bacterial cells and their growth characteristics are presented in Table 1 while the biochemical and antibiotic sensitivity tests of the bacterium are presented in Table 1, 2, respectively. Isolated bacterial strain was identified by both morphological and biochemical tests and this was further confirmed by 16S rRNA gene sequence analysis. The strain showed 100 % homology with *Klebsiella granulomatis*. The sequence was deposited in genbank with accession no-KM593690 (BankIt1760483 *Klebsiella*).

**Table 1:** Cultural characteristics and microscopic observations of the isolated bacterial strain

Agar plates	Characters	Results
Nutrient agar slant	Abundance of growth	Moderate
	Colour	Creamy White
Nutrient broth culture	Appearance	Uniform with fine turbidity
Microscopic observations	Gram staining	Gram-negative
	Motility	Motile
MacConkey	Appearance	Mucoid
	Colour	Pink
XLD	Colour	Yellow

**Table 2:** Biochemical test results for the isolated bacterial strain (*K. granulomatis*)

Biochemical test	Reaction	Sugar utilization	Reaction
Catalase	+	Glucose	+
Oxidase	+	Arabinose	−
Nitrate reduction	−	Lactose	+
Indole	−	Xylose	+
Methyl Red	−	Malonate	+
VP	+	Rhamnose	+
Lysine decarboxylase	+	Raffinose	−
Citrate	+	Glucose	+
Urease	+	Arabinose	−
H_2_S production	−	Lactose	+
β-galactosidase	+	Dulcitol	−

**(**+ = microbial growth, − = no growth**)**

### Effect of temperature and pH on bacterial growth

**Fig. 1:**
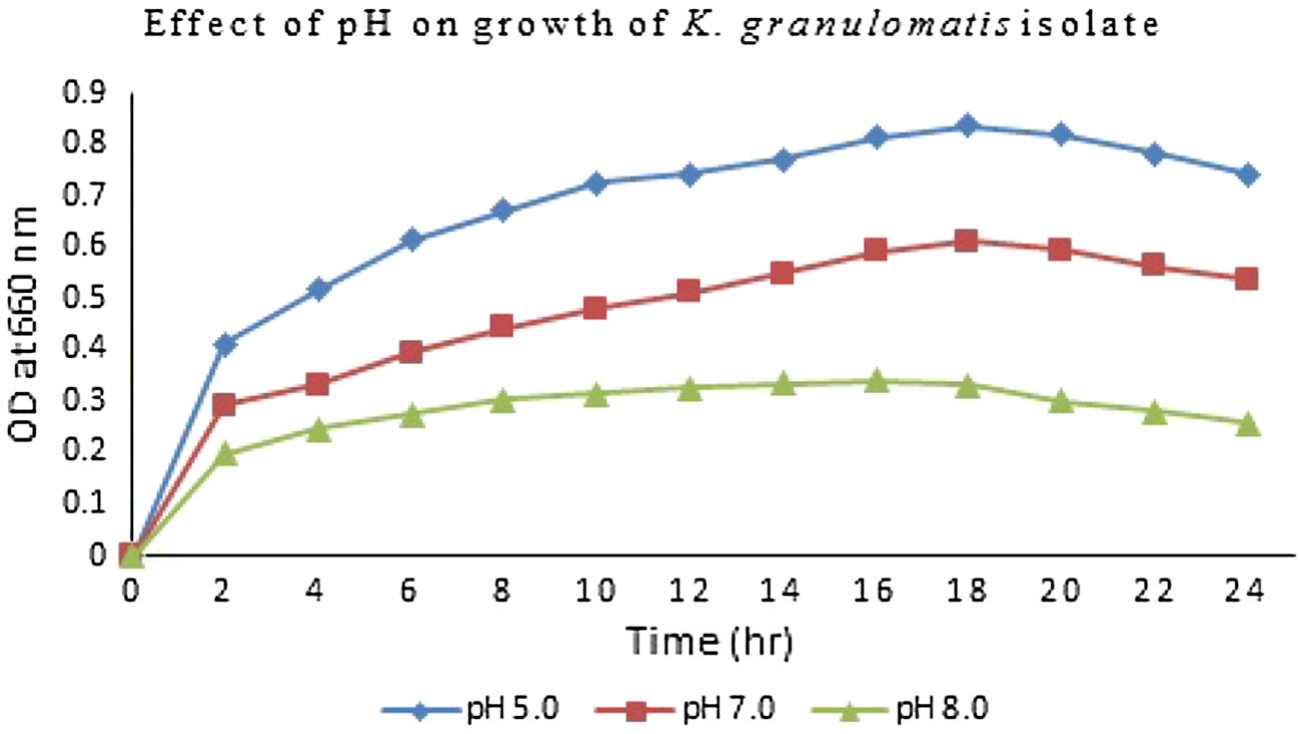
Effects of pH on bacterial growth

**Fig. 2:**
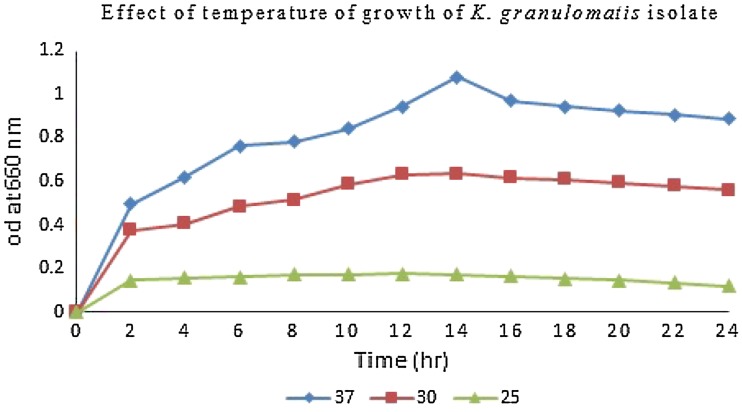
Effects of temperature on bacterial growth

To verity the effects of temperature and pH of growth medium on the growth rate of the bacterium, a series of investigations were carried out which are presented in Figs. 1, 2, respectively. The optimum pH for the growth of the isolate was 7.0 and extreme pH (5.0 and 8.0) restricted the bacterial growth (Fig. 1). The highest growth rate was observed in the bacterium. The optimum temperature for the growth of bacterium was found at 37 °C and the extreme temperatures between 30 and 25 °C restricted the bacterial growth. At 37 °C the rate of the best growth was found to be the highest (OD = 0.33) after 16 h of culture and after 18 h the OD started to decrease (Fig. 2).

### Antibiotic susceptibility of the isolate

The isolate showed resistance against seven antibiotics included in this study (Table 3). Only azithromycin, gentamycin and ciprofloxacin were found to be effective against the isolate.

**Table 3:** Antibiotic sensitivity tests

Antibiotics	Disc distance (mm)	R	S and I
Vancomycin	5	R	–
Pefloxacin	12	–	I
Cefuroxime sodium	5	R	–
Penicillin	5	R	–
Cephardine	5	R	–
Mecillinam	5	R	–
Nitro furantoin	5	R	–
Vancomycin	5	R	–
Azithromycine	18	–	S
Gentamycin	16	–	S
Ciprofloxacin	16	–	S

(5–10 mm) = Resistant to antibiotics (R); (15–20 mm) = Sensitive to antibiotic (S), (10–15 mm) = intermediate resistance (I)

### Determination of pathogenicity of the isolated bacterium

The isolate was found to be pathogenic to *Bombyx mori* as it induces bacterial flacherrie like symptoms upon being infected by the larvae. The silkworms infected with the isolate exhibited symptoms similar to those of bacterial flacherrie as described by Zhang et al. (2013). Mortality rate of the larvae was increased with increasing bacterial concentration. LC50 was found to be 2.54 × 10^7^ (Table 4).

**Table 4:** Pathogenicity of the *Klebsiella granulomatis* isolate against silkworm

Concentration of bacteria (cfu/mL)	Corrected mortality (%)	LC_50_	Pearson correlation
10^4^	21.36	2.54 × 10^7^	0.924
10^5^	37.25		
10^6^	61.33		
10^7^	92.43		

### Effect of ciprofloxacin on larvae infected with the isolate

It was found that the production of cocoon as well as the weight, length and width in the control (only bacteria, no antibiotic) and antibiotic-injected groups vary significantly (Table 5). Weight, length and width of the cocoon treated with ciprofloxacin had better weight, length and width indicating that ciprofloxacin can be used to control flacherrie caused by *K. granulomatis* as well as by other bacteria.

**Table 5:** Effect of ciprofloxacin treatment on the cocoon traits in *B. mori*

SL	Wt^a^	Wt^b^	Wt^c^	Lt^a^	Lt^b^	Lt^c^	Wd^a^	Wd^b^	Wd^c^
1	0.91	1.08	1.18	3.10	3.40	4.57	4.60	5.60	5.98
2	0.86	1.04	1.21	3.50	4.30	4.62	4.30	5.90	5.89
3	0.84	1.07	1.19	3.00	3.20	4.39	4.40	5.60	5.88
4	0.87	1.12	1.23	3.40	3.90	4.29	4.60	6.10	5.93
5	0.62	1.06	1.22	3.50	4.10	4.44	4.10	5.30	5.79
6	0.84	1.02	1.23	3.30	3.60	4.39	4.20	5.30	5.82
7	0.94	1.11	1.19	2.90	3.90	4.51	4.30	5.40	5.88
8	0.85	1.10	1.20	3.10	3.80	4.49	4.90	5.20	5.91
9	0.94	1.08	1.17	3.10	3.70	4.29	4.70	5.20	5.83
10	0.88	1.04	1.18	3.00	3.60	4.31	4.20	5.10	5.94
Mean ± SD	0.855 ± 0.09	1.07 ± 0.03	1.2 ± 0.02	3.19 ± 0.22	3.75 ± 0.32	4.42 ± 0.11	4.43 ± 0.26	5.47 ± 0.33	5.88 ± 0.05

Cocoon weights are in g, lengths and widths are in mm

*Wt* weight, *Lt* Length, *Wd* width

^a^Diseased silkworm treated with no antibiotics

^b^Diseased silkworm treated with antibiotic

^c^Non-diseased silkworm

## Discussion

The economic status of Bangladesh mainly depends on agriculture. Silk manufacturing is the traditional occupation in Rajshahi, Bangladesh. Many people earn their lively hood by silkworm rearing. During silkworm rearing, the silkworm comes into contact with various pathogenic bacteria. About 34 to 40 % of total crop in a year has been reported to be lost due to diseases like flacherie. Bacterial flacherie in silkworm is known to be caused by consortium of various pathogeinc bacteria.

In this study the microorganism was identified as a number of the genera *Klebsiella granulomatis* bacterium. Physiological and biochemical tests revealed that the microorganism was gram-negative, rod-shaped and non-motile bacterium. After a 16S rRNA gene sequencing and BLAST search, 99 % similarity was observed with *Klebsiella granulomatis*. Optimum pH and temperature for growth of the isolate was found to be 7 and 37 °C, respectively.

Many previous studies reported isolation of bacteria of different genus from diseased silkworm, such as, *Acrobacter cloacae*, *Achromobacter superficialis*, *Achromobacter delmarvae*, *Pseudomonas boreopolis,* *Pseudomonas ovalis*, *Escherichia freundii* and *Staphylococcus albus* (Chitra et al. 1973); *Bacillus subtilis*, *Bacillus cereus, Staphylococcus albus, Stabhylococcus aureus* and *Klebsiella cloacae* (Priyadharshini et al. 2008); *Streptococcus faecalis* (Patil 1994), *Bacillus thuringienisis* (Nataraju et al. 1991), *Streptococcus* spp*. Serratia* spp. and *Bacillus* spp. (Anitha et al. 1994); *Pseudomonas chlororaphis* (Tao et al. 2011) and *Providencia rettgeri* (Zhang et al. 2013). The occurrence of *Klebsiella* *granulomatis*, gram negative *bacilli* in the silkworm haemolymph is being reported for the first time through this study.

Antibiotics are used to find out their effectiveness against pathogenic bacteria (Mahmoud et al. 2012). As a result, bacteria associated with silkworm are prone to develop resistance to commonly used antibiotics. Eleven types of antibiotic were used in this study and only three antibiotics viz. azithromycine, gentamycin and ciprofloxacin were showed strongly effective against the isolated bacterium. This high antibiotic resistance of the bacterium associated with flacherrie indicates reduced usability of current antibiotics. Similar antibiotic resistance of bacteria associated with flacherrie has been reported by many previous studies (Nahar 1995, Kim et al. 2002).

Mortality rate increased with increased bacterial dose. At ~10^4^ cfu/ml bacterial dose mortality rate was 21.36 %, at ~10^5^ cfu/ml 37.25 %, at ~10^6^ cfu/ml 61.33 % and at ~10^7^ cfu/ml mortality rate was 92.43 %. LC50 was found to be 2.54 × 10^7^ with pearson correlation 0.924.

Antibiotics improve feed consumption and growth by stimulating metabolic processes within the silkworm as well as reduce the occurrence of diseases which causes immense loss to sericulture industry. It was found that ciprofloxacin significantly increases the effective rate of rearing and cocoon weights and cocoon length and width were significantly increased under the effects of antibiotic treatment comparing with control. Similar effect by gentamycin was reported by Mahmoud et al. (2012). Use of antibiotic to prevent bacterial disease of silkworm has also been reported by many studies (Hamamoto et al. 2005, Kaito et al. 2002).

Administration of antibiotics and dose of administration are critical as in many cases administration of antibiotics was reported to have detrimental effects on intestinal micro-flora of silkworms which cause adverse effects on the physiological system (Subramanian et al., 2009). As a result, it is recommended to apply low concentration of antibiotics to induce prophylactic measures to prevent bacterial infections, as also recommended by other researchers (Sheebha et al. 2008, Anandakumar et al. 2012).

## Conclusion

This study reports for the first time about bacterial flacherrie infection of silkworm in Bangladesh and *K. granulomatis* was found to be a causative bacterium of flacherrie disease. Knowledge on causative bacteria, pathogenic potential and antibiotic resistance is important to deduce an effective treatment strategy. Results of this study in combination with previous study results stress the need for more extensive research focusing on prevention and control of bacterial flacherrie disease of silkworm of Bangladesh.
